# T177. STRUCTURAL ORGANIZATION OF THE PRAXIS NETWORK PREDICTS GESTURE PRODUCTION: EVIDENCE FROM HEALTHY SUBJECTS AND PATIENTS WITH SCHIZOPHRENIA

**DOI:** 10.1093/schbul/sby016.453

**Published:** 2018-04-01

**Authors:** Petra Viher, Ahmed Abdulkadir, Katharina Stegmayer, Peter Savadijev, Marek Kubicki, Nikos Makris, Sarina Karmacharya, Andrea Federspiel, Stephan Bohlhalter, Tim Vanbellingen, René Müri, Roland Wiest, Werner Strik, Sebastian Walther

**Affiliations:** 1 University Hospital of Psychiatry Bern; 2 University Hospital of Psychiatry Bern, University of Bern; 3 Brigham and Women′s Hospital, Harvard Medical School; 4 Institute of Neuroradiology, University of Bern

## Abstract

**Background:**

Hand gestures are an integral part of social interactions and are involved in nonverbal and verbal communication. The convey language that is expressed by motor actions, and thus depend on the interplay of various brain regions. Several functional magnetic resonance imaging studies in healthy subjects suggest the praxis network for gesture production, involving distinct frontal, parietal and temporal regions. Lesions studies in subjects with apraxia, following left brain damage corroborate these findings. However, little is known about the structural connectivity underlying gesture production. We aimed to provide novel insights into the structural connectivity of the praxis network and how it is related to gesture production.

**Methods:**

Our sample consisted of 41 healthy subjects and of 40 patients with schizophrenia, demonstrating gesture impairments and structural network abnormalities. All participants performed a gesture production test, the test of upper limb apraxia and underwent diffusion weighted magnetic resonance imaging. Finsler geometry was used to investigate structural connectivity and graph theory to estimate global and local efficiency of the praxis network, which consists of 13 bilateral regions of interest.

**Results:**

Our findings showed an association of gesture production with network attributes and specific connections within the praxis network. Thus, global and local efficiency and most of the intra- and interhemispheric connections within the gesture network predicted gesture production across groups. Global efficiency of the praxis network further predicted gesture production only in the patient group. Local efficiency of many ROIs and connections of interest predicted production in patients at trend-level. In contrast, there were no significant or trend-level associations of gesture production with network attributes in controls.

**Discussion:**

The results revealed an association of impaired gesture performance with structural alterations of the praxis network, including global and local efficiency and many connections of interest. Our findings are of great importance in the understanding of the structural correlates of gesture production and shed further light on the neural underpinnings of gesture deficits in a patient group with severe social deficits.

